# Intratumor heterogeneity and T cell exhaustion in primary CNS lymphoma

**DOI:** 10.1186/s13073-022-01110-1

**Published:** 2022-09-24

**Authors:** Michael Heming, Svea Haessner, Jolien Wolbert, I-Na Lu, Xiaolin Li, Benjamin Brokinkel, Michael Müther, Markus Holling, Walter Stummer, Christian Thomas, Andreas Schulte-Mecklenbeck, Flavia de Faria, Marlon Stoeckius, Stephan Hailfinger, Georg Lenz, Kornelius Kerl, Heinz Wiendl, Gerd Meyer zu Hörste, Oliver M. Grauer

**Affiliations:** 1 Department of Neurology with Institute of Translational Neurology, University Hospital Münster, Albert-Schweitzer-Campus 1, Bldg A1, 48149 Münster, Germany; 2 The Agency for Science, Technology and Research (A*STAR), Singapore, Singapore; 3 Department of Neurosurgery, University Hospital Münster, Münster, Germany; 4 Institute of Neuropathology, University Hospital Münster, Münster, Germany; 5 Department of Pediatric Hematology and Oncology, University Hospital Münster, Münster, Germany; 6 New York Genome Center, New York, NY USA; 7 Department of Medicine A, Hematology, Oncology, and Pneumology, University Hospital Münster, Münster, Germany

**Keywords:** Primary central nervous system lymphoma, Single-cell RNA sequencing, Intratumoral heterogeneity, T cell exhaustion, Spatial transcriptomics, Flow cytometry

## Abstract

**Background:**

Primary central nervous system lymphoma (PCNSL) is a rare lymphoma of the central nervous system, usually of diffuse large B cell phenotype. Stereotactic biopsy followed by histopathology is the diagnostic standard. However, limited material is available from CNS biopsies, thus impeding an in-depth characterization of PCNSL.

**Methods:**

We performed flow cytometry, single-cell RNA sequencing, and B cell receptor sequencing of PCNSL cells released from biopsy material, blood, and cerebrospinal fluid (CSF), and spatial transcriptomics of biopsy samples.

**Results:**

PCNSL-released cells were predominantly activated CD19^+^CD20^+^CD38^+^CD27^+^ B cells. In single-cell RNA sequencing, PCNSL cells were transcriptionally heterogeneous, forming multiple malignant B cell clusters. Hyperexpanded B cell clones were shared between biopsy- and CSF- but not blood-derived cells. T cells in the tumor microenvironment upregulated immune checkpoint molecules, thereby recognizing immune evasion signals from PCNSL cells. Spatial transcriptomics revealed heterogeneous spatial organization of malignant B cell clusters, mirroring their transcriptional heterogeneity across patients, and pronounced expression of T cell exhaustion markers, co-localizing with a highly malignant B cell cluster.

**Conclusions:**

Malignant B cells in PCNSL show transcriptional and spatial intratumor heterogeneity. T cell exhaustion is frequent in the PCNSL microenvironment, co-localizes with malignant cells, and highlights the potential of personalized treatments.

**Supplementary Information:**

The online version contains supplementary material available at 10.1186/s13073-022-01110-1.

## Background

Primary central nervous system lymphoma (PCNSL) represents a rare and highly aggressive form of extranodal malignant lymphoma with an incidence of approximately 0.47 per 100,000 person-years manifesting in the craniospinal axis without clinical evidence of systemic involvement [1]. First-line therapy in PCNSL usually comprises high-dose methotrexate in combination with additional agents, but the optimal treatment regimen, especially in relapse, remains subject of ongoing studies [2]. Scarce biomaterial is available for scientific purposes because the diagnostic gold standard in PCNSL is a stereotactic central nervous system (CNS) biopsy that returns minuscule tissue pieces. The little available material is required for diagnostic histopathology, which necessitates formalin fixation and paraffin embedding, impeding transcriptional studies. The majority of PCNSL cases are histologically classified as diffuse large B cell lymphoma (DLBCL) [3] and are of an activated B cell (ABC)/non-germinal center B cell (GCB) subtype (~80%) [4].

Cerebrospinal fluid (CSF) exhibits specific alterations in PCNSL, such as elevated levels of CXCL13, IL-10, or microRNA-21 [5–7], and might therefore represent a potential surrogate when combined with high-resolution techniques. However, lymphoma cells can only be detected infrequently (13.3–23.3%) in the CSF of affected patients [8], thus further complicating mechanistic understanding and diagnosis.

Genome-wide studies revealed recurrent alterations in PCNSL that are not shared with systemic DLBCL, suggesting a distinct pathogenesis [9]. Activation of NF-kB, MYD88, BTG2, and PIM1 has been implicated in the PCNSL pathogenesis [10] and is associated with changes of the tumor microenvironment (TME) [11]. Genomic alterations may also affect genes that are involved in immune evasion mechanisms, including deletions in the *HLA* locus (6p21), copy-number loss of *B2M* (15q21.2), or copy-number gain of *CD274*/PD-L1 (9p24.1) [12]. Accordingly, immune checkpoint molecules (PD-1, CTLA-4, TIM-3) have emerged as potential therapeutic targets in PCNSL [13]. Prospective trials of the checkpoint blockade with PD-1 inhibitors have been initiated in PCNSL (NCT02779101, NCT02857426), but no results have been published yet. A small retrospective study reported long-term responses in five of six patients following nivolumab treatment [14]. Despite these advances, the TME in PCNSL remains poorly defined [11].

Single-cell RNA-sequencing (scRNA-seq) is a powerful tool to uncover intratumor heterogeneity, for example in peripheral B cell lymphomas [15]. Malignant subpopulations in peripheral lymphomas were transcriptionally distinct with specific drug response profiles, potentially paving the way for personalized cancer treatments [15]. Here, we circumvented the problem of limited sample access by studying cells released into the surrounding liquid from a CNS biopsy rather than the biopsy itself and denoted this approach “Whiskey Method.” Flow cytometry of such released cells facilitated immediate distinction between lymphomatous and non-lymphomatous tissue biopsies. By combining scRNA-seq and spatial transcriptomics, we identified a surprising intratumoral transcriptional heterogeneity with distinct spatial patterns of malignant B cell clusters. Immune repertoire analysis displayed shared hyperexpanded clones between biopsy- and CSF-, but not blood-derived cells. The TME was characterized by increased expression of immune checkpoint molecules, immune evasion signaling from the PCNSL, and spatially defined T cell exhaustion, which co-localized with a highly malignant B cell cluster. We thus identify a potential novel avenue for detection, characterization, and ultimately treatment of rare, but aggressive PCNSL.

## Methods

### Patient recruitment, follow-up, and therapy

We recruited sixteen patients with radiological findings suggestive of PCNSL in the University Hospital Münster. Patients received a stereotactic biopsy by the neurosurgeon to confirm the diagnosis and specify the lymphoma type for further treatment. Eight of the sixteen patients (mean age 74 years, M:F ratio 7:1) yielded a positive diagnosis of DLBCL of the CNS whereas eight patients (mean age 68 years, M:F ratio 6:2) were diagnosed with glioblastoma. Multiparameter flow cytometry of PBMCs was performed in 14 of 16 cases, and of biopsy fluid in all cases. scRNA-seq was applied to five samples of two PCNSL patients (PBMC, biopsy fluid, and CSF of patient 1; PBMC and biopsy fluid of patient 2). Spatial transcriptomics was performed on biopsy material obtained from 4 PCNSL patients (patients 1–3 and patient 7). More details are given in Additional file 1: Table S1.

### The “Whiskey Method”

**Fig. 1:**
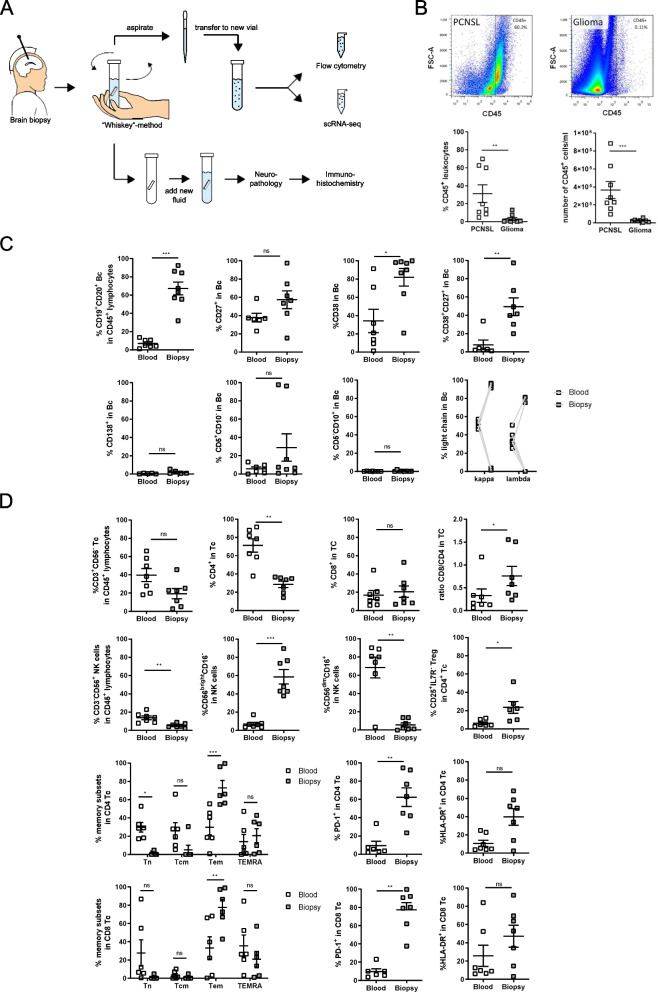
The “Whiskey Method” provides unique access to malignant cells and facilitates detection of PCNSL. **A** Experimental scheme of the “Whiskey Method.” Brain biopsy was collected into a sterile sampling tube filled with 2 ml 0.9% sodium chloride. The sample container was swirled for 10 s, and the supernatant was immediately subjected to flow cytometric and scRNA-seq analyses. The biopsy tissue was sent for neuropathological evaluation. **B** Representative flow cytometry dot plots of CD45^+^ leukocytes and flow cytometry analysis of the percentage and number of CD45^+^ leukocytes in the samples of PCNSL (*n* = 8) and glioblastoma (*n* = 8) patients (Additional file 1: Table S1 and Additional file 2: Supplementary Methods). **C** Flow cytometry analysis of CD19^+^CD20^+^ B cells (Bc) within the CD45^+^ leukocyte gate in peripheral blood (*n* = 7) and biopsy samples (*n* = 8) of PCNSL samples. B cell subpopulations were further characterized by analyzing the distribution of CD27^+^CD20^+^, CD38^+^, CD38^+^CD27^+^, CD138+, CD5^+^CD10^−^, CD5^−^CD10^+^, and of Ig kappa or lambda light chain expressing cells. The gating strategy is shown in Additional file 4: Fig. S1. **D** Flow cytometry analysis of CD3^+^CD56^-^ T cells (Tc) and CD3^−^CD56^+^ NK cells in the CD45^+^ lymphocytes gate within peripheral blood (*n* = 7) and biopsy samples (*n* = 7) are shown. NK cells were further subclustered into CD56^dim^CD16^+^ and CD56^bright^CD16^−^ NK cells. T cells were further characterized by the percentage and ratio of CD4^+^ and CD8^+^ Tc, and CD4^+^CD25^+^IL7R^−^ Treg, as well as the distribution of naive T cells (Tn; CCR7^+^CD45RA^+^), central memory T cells (Tcm; CCR7^+^CD45RA^-^), effector memory T cells (Tem; CCR7^−^CD45RA^−^), effector memory recently activated T cells (TEMRA; CCR7^−^CD45RA^+^). In addition, percentages of CD4^+^ and CD8^+^ T cells expressing HLA-DR and PD-1 were determined. Gating strategies are depicted in Additional file 4: Fig. S2. Data are depicted as mean, error bars show the SE; two-sided Mann-Whitney *U* test was used to calculate statistical significance between groups; * *p*<0.05, ** *p*<0.01, *** *p* <0.001, **** *p* <0.0001, ns not significant

Samples were obtained using a frame-based (Leksell Stereotactic System, Elekta Instrument AB, Stockholm, Sweden) or a frameless image-guided (VarioGuide, Brainlab AG, Munich, Germany) stereotactic system. In all cases, the target point was defined within the contrast-enhancing, non-necrotic lymphoma-suspicious lesion (Fig. 1A). After reaching the target point with the biopsy needle, samples were gained by careful aspiration using a 2-ml syringe filled with 1 ml sterile 0.9% sodium chloride solution. The obtained tissue was subsequently transferred into a sterile sample container filled with 2 ml 0.9% sodium chloride. Suspension for cytometry and scRNA-seq was prepared after the first successful aspiration of tissue to avoid potential contamination by blood cells after repeated biopsies. The cell suspension was prepared by gently shaking the sample container for 10 s. The supernatant was subsequently transferred to a second sample container and subjected to flow cytometric and scRNA-seq analyses, while the biopsy tissue remained in the first sampling tube and was complemented by additional samples before being sent for neuropathological evaluation. Immediately after collection, cell viability was determined by trypan blue staining, which was higher than 95% (97±1.8%) in our samples. Samples were spun down and cell pellets were resuspended in 1 ml PBS. One hundred microliters of the cell suspension (1:10 dilution) was used for FACS staining (see “Methods”). After staining and washing, cells were resuspended in 200 μl FACS buffer (PBS, 1% FCS, 2 mM EDTA) and the whole volume of the sample was acquired by flow cytometry. Total cell number of CD45+ leukocytes was determined by counting all cells within the CD45^+^ gate × dilution factor (10x) (Fig. 1A).

### Multiparameter flow cytometry

PBMCs and biopsy fluid samples were stained for 30 min at 4 °C using pre-defined panels of directly fluorochrome-conjugated monoclonal antibodies (mAbs) at a working concentration of 5–10 μg/mL (see Additional file 2: Supplementary Methods for more information on the gating, and Additional file 3: Table S2 for the list of flow cytometry antibodies). After washing, all samples were analyzed using the Navios^TM^ or CytoFlex^TM^ flow cytometer (Beckman Coulter, Germany) and the FlowJo^TM^ Software V10. The gating strategies are depicted in Additional file 4: Fig. S1-3.

### Histology and immunohistochemistry

Immunohistochemical staining of formalin-fixed and paraffin-embedded tissue for CD3 (rabbit polyclonal, #GA503, Dako, Glostrup, Denmark), CD20 (mouse monoclonal, #GA604, Dako, Glostrup, Denmark), CD79a (mouse monoclonal, #GA621, Dako, Glostrup, Denmark), and Mib1/Ki67 (mouse monoclonal, #GA626, Dako, Glostrup, Denmark) was performed using the streptavidin-biotin method on an automated staining system (Omnis, DAKO). Slides were counterstained with hematoxylin.

### Generation of single-cell libraries, sequencing, and preprocessing of sequencing data

The samples were loaded onto the 10x Genomics Chromium Single Cell Controller, using the Chromium Single Cell 3′ Library & Gel Bead Kit v2-3. B cells were positively selected from blood-derived cells, using CD20-microbeads according to the manufacturer’s protocol (Miltenyi). Biopsy-derived cells and B cell-enriched blood cells in patient 1 were processed using CITE-seq supported scRNA-seq, all other samples were processed with standard scRNA-seq. Sequencing was carried out commercially on an Illumina Nextseq 500 with a 26-8-0-57 read setup, a Nextseq 2000 with a 28-8-0-91 read setup, and a Novaseq 6000 with 150-8-0-150 read setup. Counting of CITE-seq data was performed with *CITE-seq-count* [16]. To generate spliced/unspliced expression matrices, velocyto v0.17 [17] was employed with the run10x command, using the gene annotation file from cellranger and the human repeat masker file from the UCSC genome browser. For further details, see Additional file 2: Supplementary Methods and Additional file 5: Table S3.

### Reconstructing BCR information from 3′ scRNA-seq libraries

To obtain single-cell BCR repertoire information, a novel method to sequence antigen receptor information from 3′ scRNA-seq libraries was used, which we have described previously [18]. The method allows shortening the constant region of antigen receptors during enrichment while maintaining their cell barcode and unique molecular identifier (UMI) information attached to the 3′ of the cDNA molecules. In summary, the method involves self-circulating the cDNA library, enriching the VDJ region, and re-linearizing. For more details, see Additional file 2: Supplementary Methods.

### Spatial transcriptomic investigation

Five-micrometer FFPE sections of human PCNSL samples were placed on a Visium Gene Expression (GEX) slide (10x Genomics), which incorporates about 5000 molecularly barcoded and spatially encoded capture spots. Deparaffinization, hematoxylin and eosin (H&E) staining, and decrosslinking were performed according to the protocol of Visium Spatial Gene Expression for FFPE (10x Genomics). After incubation with the Probe Hybridization Mix (10x Genomics), the tissues were permeabilized and the representative probes were captured. GEX libraries were generated for each section and then sequenced on an Illumina NextSeq2000. The data were processed by *spaceranger count* v1.3 together with the corresponding H&E-stained images in tiff format to generate the gene expression matrices (default settings). We used the Human Probe Set from 10x Genomics (Visium Human Transcriptome Probe Set v1.0) to map the data. For further technical details, see Additional file 5: Table S3.

### Data analysis of scRNA-seq

Downstream analysis was mainly performed with the R package Seurat v4.1 [19], following the official tutorial and as described previously [18, 20]. Shortly, low-quality cells and cell doublets were removed by filtering cells with few genes (<200), high number of genes (>3500–6000), or high mitochondrial percentages (>20–30%) for each sample separately. Doublets were removed with scDblFinder. scRNA-seq data were normalized with LogNormalize in Seurat (scale factor 10,000), while CITE-seq data were normalized with the centered log ratio method. Highly variable genes were identified and data were scaled. We used principal component analysis (PCA) for primary dimensionality reduction. To identify the number of PCs for further analysis, we performed an elbow plot and used 30 dimensions for downstream analysis. Batch effects were addressed for each sample separately with Harmony v0.1 [21]. Clusters were identified by the FindNeighbors (based on KNN graphs) and FindClusters (based on Louvain method, resolution = 0.2) functions in Seurat. Harmony embeddings were used as input for Uniform Manifold Approximation and Projection (UMAP), which allows data visualization in a two-dimensional space. Clusters were annotated based on known marker genes. B and T cell clusters were extracted as separate subsets, and PCA (based on the top 2000 highly variable features), batch correction with Harmony, UMAP, and clustering with Seurat were re-performed on each subset. DotPlots and FeaturePlots were generated with internal visualization functions in Seurat. Heatmaps were created with pheatmap v1.0, stacked bar plots, and enrichment dotplots were generated with ggplot2 v3.3. Differentially expressed (DE) genes were calculated with the FindMarker function in Seurat (RunPresto implementation) based on the Wilcoxon rank sum test with an adjusted *p*-value threshold (based on Bonferroni correction) of 0.05, minimum fraction of 10%, and average log2 fold change of 0.25. Differentially expressed genes were plotted in volcano plots, using Enhanced Volcano v1.12. We used the EnrichR package v3.0 [22] to perform the Enrichment analysis based on the NCI-Nature Pathway Interaction Database. Comparisons of our scRNA-seq dataset with data from Roider et al. [15] were made with clustifyr v1.6 [23] and were based on annotations provided by the authors. T cells were projected onto the UMAP embeddings of a murine reference atlas with ProjecTILs [24], using default settings. Copy number variations were determined with infercnv v1.10 [25]. The B cell clusters were downsampled to 1000 cells per cluster, GENCODE v19 was used as a gene order file, and the cells from the nmBc1/2 clusters were used as reference cells. Infercnv was run with a cutoff of 0.1, as advised in the official vignette for 10x data, denoising by the default dynamic thresholding and an additional median denoising filter. The chromosomal aberrations were added to the Seurat object with infercnv and visualized as a feature plot with Seurat.

### RNA velocity and pseudotime analysis

RNA velocity analysis was carried out with scVelo v.0.2.5 [26], following the official tutorial. We used slingshot v2.2 [27] to perform the pseudotime analysis. Reclustered cells from mBc1-4 clusters were imported into *scVelo*. Data were normalized and logarithmized (using the top 5000 genes), and first- and second-order moments were computed (with 30 PCs and 30 neighbors). Finally, RNA velocity was calculated, using the generalized dynamical model, which is solved in a likelihood-based expectation-maximization framework to learn the unspliced/spliced phase trajectories for each gene.

Slingshot was run with the reclustered cells from mBc1-4 clusters, with mBc2 as a starting cluster and default parameters. The pseudotime lineages were visualized with the UMAP embeddings from Seurat using ggplot2.

### Identifying cellular interactions

We analyzed cellular signaling with CellPhoneDB v3.0 [28]. Processed scRNA-seq data of biopsy-derived cells including manual cluster annotations were used as input. Statistical iterations were set to 1000 and ligands/receptors expressed by less than 1% of the cells were removed. The resulting ligand-receptor pairs are based on the CellPhoneDB repository.

### Single-cell immune repertoire analysis

Single-cell BCR data were analyzed with scRepertoire v1.5 [29] according to the official vignette. BCR heavy and light chains were combined based on their cellular barcodes. Cells with a missing chain or more than two immune receptor chains were removed. scBCR were merged with scRNA-seq data of the B cell clusters. The categorization into clone types was based on the amino acid sequence.

### Spatial transcriptomics analysis

Visium data were analyzed with Seurat v4.1 [19] following the official tutorial. We normalized the data with sctransform [30]. To integrate the scRNA-seq results, we used the “anchor”-based method in Seurat.

### Statistics

Statistical analysis was performed using Mann-Whitney *U* test and, if applicable, with Bonferroni adjustment for multiple hypothesis testing.

## Results

### The “Whiskey Method”: a simple tool for accelerated detection and characterization of PCNSL

Little material is available from CNS biopsies, and it is required for diagnostic confirmation of suspected PCNSL by histology and immunohistochemistry. Here, we identified a simple, but efficient way to obtain suspended PCNSL cells that were abundantly released into the surrounding liquid while briefly swirling the transferred biopsy material in saline. Due to the swirling movement, we denominated this approach the “Whiskey Method” (Fig. 1A). The method did not compromise the quality of the histopathology of the biopsy (Additional file 4: Fig. S4A,B). In total, we obtained biopsy-derived cells from sixteen patients and immediately performed flow cytometry for diagnostic purposes and evaluation of the TME (Additional file 1: Table S1). None of those patients had received corticosteroids or chemotherapy before collecting biopsy-derived cells (Additional file 1: Table S1). FACS analyses of cells released from the biopsy revealed that the mean percentage and number of CD45^+^ leukocytes was >10 times higher in samples from patients subsequently histologically diagnosed as PCNSL (*n* = 8, *M* = 31.3%, SEM = 9.8%; *M* = 366,094 cells, SEM = 94,908 cells) than in glioblastoma patients (*n* = 8, *M* = 3.1%, SEM = 1.7%; *M* = 24,353 cells, SEM = 5758 cells) (Fig. 1B). Tumor cells detach from the tumor bulk more easily in PCNSL than in glioblastoma patients, likely because of the discohesive growth pattern in PCNSL and less branched morphology compared to glioma cells (Additional file 4: Fig. S4C,D). In addition, the majority of biopsy-released CD45^+^ cells within the PCNSL samples consisted of CD19^+^CD20^+^ B cells (Bc) (*M* = 67.3%, SEM = 7.0%), while Bc proportions in blood (*M* = 7.0%, SEM = 1.7%) were in the expected range (Fig. 1C, Additional file 4: Fig. S1).

Extended flow cytometry showed that CD19^+^CD20^+^ Bc obtained from PCNSL biopsies were CD10 negative, showed variable expression of CD5 and high CD27 expression (Fig. 1C, Additional file 4: Fig. S1). Both CD38^+^ and CD38^+^CD27^+^ Bcs were significantly elevated in biopsy-derived Bcs when compared to blood, while CD20^+^CD138^+^ plasma cells were not increased. Immunoglobulin (Ig) light chain restriction (>80% expression of Kappa (*κ*) or Lambda (*λ*) Ig) was detected on all biopsy-derived Bcs. In peripheral blood, *κ*/*λ* ratios were mostly within the expected range (*M* = 1.57) [31]. The “Whiskey Method” thus facilitates the detection and further characterization of PCNSL cells within a few hours.

### Distinct immune cell alterations in the PCNSL microenvironment

Next, we aimed to characterize the TME by evaluating immune cells infiltrating PCNSLs by flow cytometry (Fig. 1D, Additional file 4: Fig. S2). Biopsy material contained lower proportions of CD4^+^ T cells (Tc) than blood. Similar percentages of CD8^+^ Tc were found in peripheral blood and biopsies, leading to an increase in the CD8/CD4 ratio at the tumor site. Tumor-infiltrating CD4^+^ and CD8^+^ Tc displayed an effector memory phenotype (Fig. 1D). Moreover, we identified elevated proportions of CD4^+^CD25^+^IL7R^−^ regulatory Tc (Tregs) within the biopsy material. CD3^−^CD56^+^ NK cells infiltrated the tumor at low frequencies with a predominance of CD56^bright^CD16^dim^ NK cells, whereas peripheral blood was dominated by CD56^dim^CD16^+^ NK cells. Finally, we detected an induction of the immune checkpoint molecule PD-1 on CD4^+^ and CD8^+^ Tc in the biopsy. Collectively, we identified a distinct cellular composition of the TME in PCNSL, featuring signs of T cell exhaustion.

### Single-cell transcriptomics reveals heterogeneity of malignant B cells in PCNSL

We sought to better characterize PCNSL by combining the “Whiskey Method” with single-cell RNA sequencing (scRNA-seq). We applied scRNA-seq to five samples from two patients with PCNSL (patients 1 and 2, Additional file 1: Table S1). We performed scRNA-seq and single-cell B cell receptor sequencing (scBCR) of cells from biopsy and peripheral blood (Bc-enriched using anti-CD20 microbeads) at the time of stereotactic biopsy from both patients (Additional file 4: Fig. S5). In addition, we collected CSF in patient 1 at relapse and also performed CITE-seq in blood and biopsy material from this patient (Additional file 1: Table S1).

**Fig. 2:**
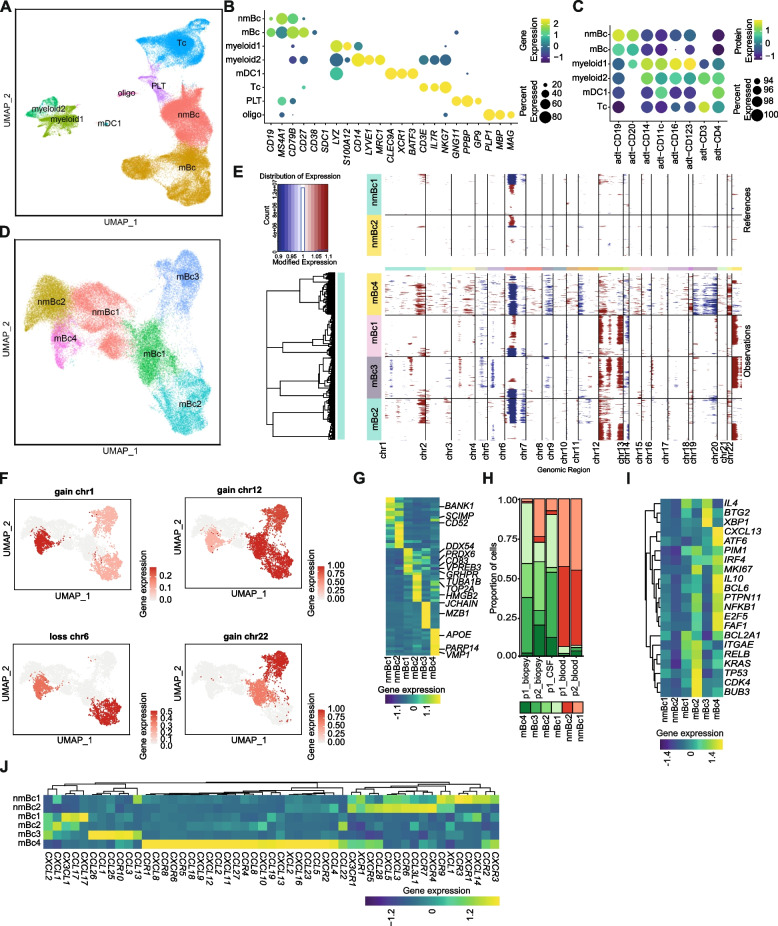
Single-cell transcriptomic reveals heterogeneous malignant B cell phenotypes in PCNSL. **A** UMAP plot of 73,896 total single-cell transcriptomes aggregated from five samples (patient 1: biopsy, blood, CSF; patient2: biopsy, blood). **B** Gene and protein (**C**) cell markers of the clusters identified by single-cell RNA sequencing (scRNA-seq) and CITE-seq (biopsy and blood from patient 1). Color encodes average gene/protein expression, and dot size represents the percentage of cells expressing the gene. The threshold of percentage of cells expressing the gene/protein was set to 15% in B and 90% in **C**. **D** UMAP plot of 45,890 reclustered B cells (mBc and nmBc cluster from A). **E** Analysis of copy number variations of downsampled B cell clusters. The nmBc1-2 clusters were used as reference cells and mBc1-4 as observations. The amplification of chromosomal regions is colored in red and the deletion of chromosomal regions in blue. **F** Feature plots of chromosomal gains and losses with the UMAP embeddings of **D**. Color encodes the proportion of chromosomal aberration. **G** Top ten differentially expressed genes of each B cell cluster shown in a heatmap. Selected genes are highlighted. Gene expression values were scaled gene-wise. **H** Proportions of B cells split by sample and colored by cluster name. **I–J** Gene expression heatmap of known PCNSL- associated genes (**I**) and of chemokines and their receptors (**J**) in B cell clusters, scaled gene-wise. Gene name - alias: *MS4A1* - *CD20*; *SDC1* - *CD138*; *SELL* - *CD62L*; *IRF4* - *MUM1.* Abbreviations: mBc - malignant B cells; nmBc - non-malignant B cells; mDC1 - myeloid dendritic cells type 1; oligo - oligodendrocytes; Tc - T cells; PLT -platelets; p1 - patient 1; p2 - patient 2; CSF - cerebrospinal fluid

We merged the data from all samples with batch correction and thereby obtained 73,896 total single-cell transcriptomes (biopsy = 36,266; blood = 33,342; CSF = 4288) after removing low-quality cells and doublets (Additional file 5: Table S3, Methods). We annotated the main clusters based on the expression of marker genes (Fig. 2A,B). Two large clusters expressed Bc markers and were tentatively named non-malignant Bc (nmBc) and malignant Bc (mBc) (*CD19*, *MS4A1*/*CD20*, *CD79B*) (Fig. 2B, Additional file 6: Table S4). In accordance with flow cytometry, *CD27* and *CD38* expressions were increased in mBc and *SDC1/*CD138 was absent, as it is well known in PCNSL [32] (Fig. 2B). Furthermore, we identified two myeloid clusters with monocyte and granulocyte markers (myeloid1-2: *LYZ*, *S100A12*, *CD14*, *LYVE1*, *MRC1*) and a cluster exhibiting mDC1 markers (mDC1: *CLEC9A*, *XCR1*, *BATF3*). Furthermore, we detected a T/NK cell cluster (Tc: *CD3E*, *TRAC*, *IL7R*, *NKG7*), a platelet cell cluster (PLT: *CLU*, *GNG11*, *PPBP*, *GP9*), and an oligodendrocyte cluster (oligo: *PLP1*, *MBP*, *MAG*). We confirmed the identity of the main cell populations on the protein level by using CITE-seq in one patient (Fig. 2C).

To better understand the intratumor heterogeneity of PCNSL, we investigated the B cell clusters in more detail by subclustering all cells in the mBc and nmBc clusters (Fig. 2D). We identified four clusters annotated as malignant clusters (mBc1-4) that showed chromosomal aberrations commonly found in PCNSL [33], including gains in chromosome 1, 12, and 22, and losses in chromosome 6 (Fig. 2D–F). This does not imply that all cells of the respective clusters are necessarily malignant. We visualized the most differentially expressed genes between the Bc clusters (Fig. 2G; Additional file 7: Table S5). The mBc1 cluster expressed the pre-B cell receptor-associated molecule *VPREB3*, the B cell activation marker *CD83*, and genes associated with cell metabolism, cellular growth, and tumor progression (*DDX54*, *PRDX6*, *GRHPR*). We found an immature, dedifferentiated phenotype with a distinct expression of cell cycle (*TOP2A*, *HMGB2*, *TUBA1B*) and proliferation genes (*MKI67*) in mBc2 (Fig. 2G). The mBc3 cluster was characterized by a more mature phenotype with signs of class-switching (*JCHAIN*, *MZB1*) (Fig. 2G; Additional file 7: Table S5). We found expression of genes involved in cancer proliferation (*PARP14*, *VMP1, APOE*) in the mBc4 cluster (Fig. 2G; Additional file 7: Table S5). In contrast, nmBc resembled naive mature B cells (*CD52*, *SCIMP*, *BANK1*) as expected for blood-derived Bc (Fig. 2G; Additional file 7: Table S5). In accordance, mBc1-4 were nearly exclusively found in biopsy- and CSF-derived leukocytes, while nmBc mainly originated from blood (Fig. 2H). CSF mirrored the relative cluster abundance of the biopsy, while blood Bc featured distinct cluster proportions (Fig. 2H). The relative abundance of malignant Bc clusters was surprisingly similar across both patients in blood- and biopsy-derived leukocytes (Fig. 2H). Compared to nmBc, mBc1-4 expressed transcripts previously commonly detected in PCNSL [5, 34], lending further support to the assumed neoplastic identity of those clusters (Fig. 2I). Of note, most of those genes were differentially expressed in two of the malignant clusters (e.g., mBc2: *BUB3*, *KRAS*, *TP53*; mBc3: *XBP1*, *BTG2*; mBc4: *CXCL13*, *BCL6*, *IL10*). We thus detected a surprisingly pronounced intratumor heterogeneity in PCNSL. Based on the chromosomal aberrations, tissue origin, transcriptional profile, and presence/absence of a hyperexpanded clone (see below), we annotated mBc1-4 as malignant and nmBc as non-malignant Bc clusters.

### Differential expression of chemokines in malignant B cell clusters

Gene expression analysis of chemokines and their receptors further supported intratumoral heterogeneity of PCNSL (Fig. 2J). We observed increased expression of *CCL17*, *CXCL17,* and *CX3CL1* in mBc1, *CXCL1* in mBc2, *CCL1*, *CCL3*, *CCL25*, and *CCL26* in mBc3, and *CCL2*, *CCL5*, *CCL19*, *CCL27*, *CXCL8*, *CXCL12*, and *CXCL13* among others in mBc4 (Fig. 2J). This is in line with previous studies demonstrating that *CXCL13* is highly specific for PCNSL [5, 35]. The chemokines expressed in mBc1-4 have the potential to attract a range of immune cells, including regulatory T cells (Tregs), macrophages, neutrophils, myeloid-derived suppressor cells (MDSC), different T helper, and DC subsets (see Additional file 8: Table S6 for details) [36]. In contrast, nmBc1-2 expressed a different set of chemokines and chemokine receptors than mBc1-4 including *CXCL3*, *CXCL5*, *CCR3*, *CCR7*, *CCR9*, *CXCR1*, *CXCR4*, and *CXCR5*. Since the CXCL13–CXCR5 axis is pivotal in recruiting Bc [37], non-malignant Bc might have been attracted from the periphery to the tumor by malignant Bc during lymphoma progression.

### Malignant B cell clusters show a phenotypic gradient with multiple developmental trajectories

**Fig. 3:**
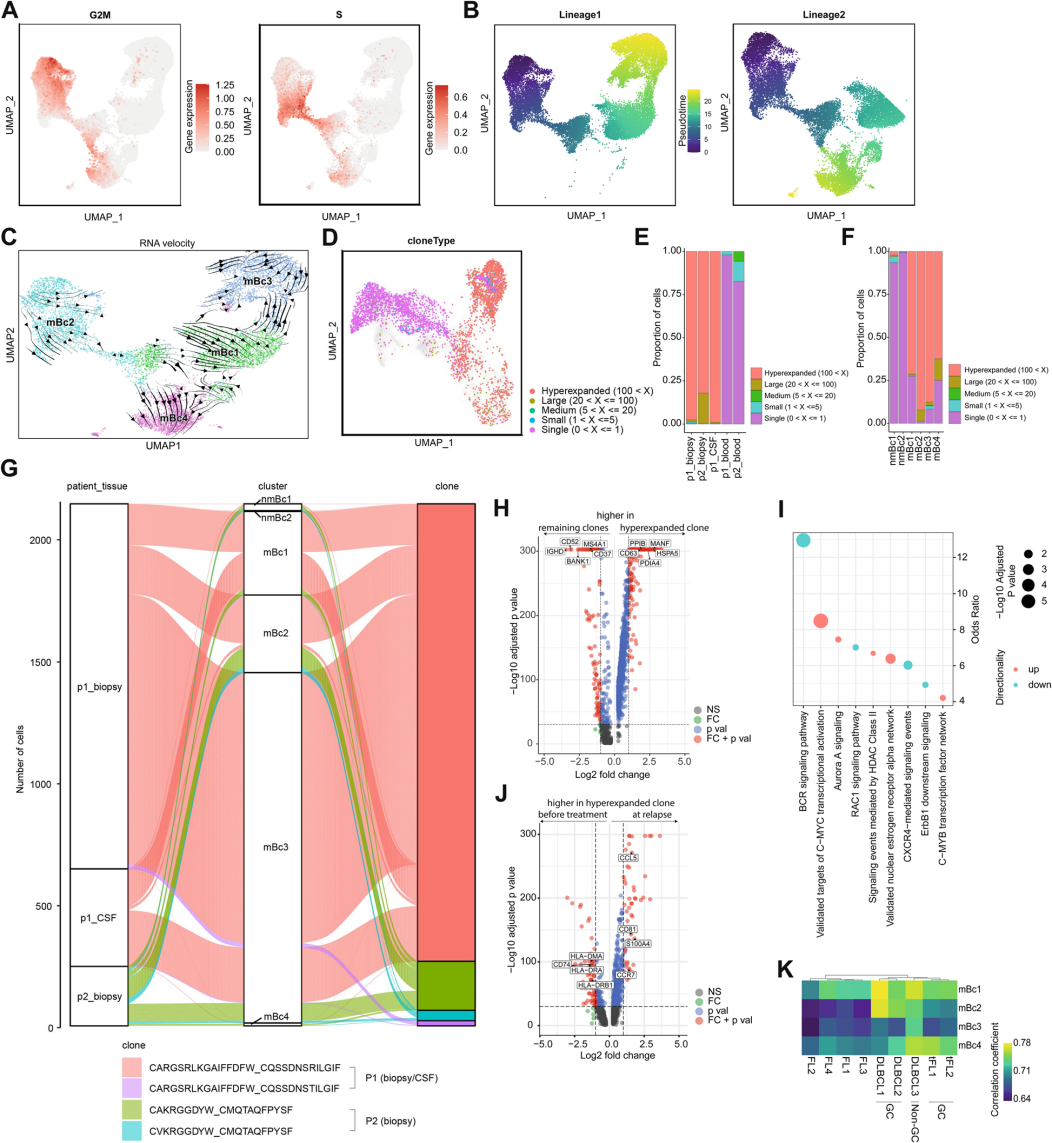
Biopsy- and CSF-derived cells but not blood cells share hyperexpanded B cell clones. **A** Geneset feature plot of G2/M and S phase with the UMAP embeddings of **C**. Color encodes gene expression. **B** Feature plots of two lineages of pseudotime analysis. Pseudotime is color-coded and the UMAP embeddings refer to **C**. **C** RNA velocity analysis of mBc1-4 clusters (Fig. 2A). Streamlines represent vector velocity fields, which show the developmental pathways. **D** UMAP plot of all B cell clusters (45,890 cells) from 5 samples (patient 1: biopsy, blood, CSF; patient 2: biopsy, blood) with color-coded frequency of B cell clones. Cells colored in transparent grey represent cells with missing BCR information. **E, F** Proportions of B cells with BCR information split by sample (**E**) or cluster (**F**) and colored by frequency. Frequency was defined by the number of B cells expressing a unique clone (paired BCR heavy and light chains). **G** Alluvial plot shows the origin tissue and cluster of the hyperexpanded clones. **H, J** Volcano plots of the differentially expressed (DE) genes of the hyperexpanded clones versus all remaining clones (**H**) and DE genes of the hyperexpanded clone of patient 1 after treatment at relapse (CSF-derived) versus before treatment (biopsy-derived) (**I**). The threshold for the log_2_ fold change was set to 1 and the threshold for the negative log_10_*p*-value to 30. **I** Significantly enriched terms of DE genes based on the NCI-Nature Pathway Interaction Database corresponding to H. Size encodes the significance and color encodes whether the term was enriched in genes with elevated or reduced gene expression. **K** Correlation coefficients between gene expression of mBc1-4 clusters (row) and different lymphomas (column) from Roider et al. [15] including four follicular lymphoma (FL1-4), four GC-derived DLBCLs, of which two were transformed from FLs (DLBCL1, DLBCL2, tFL1, and tFL2) and one non-GC-derived DLBCL (DLBCL3), visualized in a heatmap. High correlation coefficients, colored in yellow, indicate a high transcriptional overlap. Abbreviations: mBc - malignant B cells; nmBc - non-malignant B cells; p1 - patient 1; p2 - patient 2; Bc - B cells; CSF - cerebrospinal fluid; NS - not significant; FC - fold change; p val - *p*-value; DLBCL - diffuse large B cell lymphoma; FL - follicular lymphoma (FL); tFL - transformed FL

We next aimed to better understand the developmental relationship between the individual biopsy-derived Bc clusters. mBc2 displayed high expression of cell cycle genes, suggesting an immature, proliferating phenotype (Fig. 3A). We performed RNA velocity of single cells, which is based on the ratio of spliced/unspliced mRNA, with scVelo [26], a likelihood-based dynamical model, and with pseudotime (Fig. 3B,C). The resulting streamlines delineated developmental paths from mBc2 to mBc4 and from mBc2 over mBc1 to mBc3 (Fig. 3C). This suggests multiple developmental trajectories within malignant Bc, potentially differentiating from cycling and immature Bc into later Bc stages. Because of limited applicability of RNA velocity in cancer, such as absence of ancestral cells and aberrant splicing caused by mutations [38], the results should be interpreted with caution. Combining the results of the chromosomal aberration (Fig. 2E, F) with the developmental paths (Fig. 3B, C), it is noticeable that some chromosomal aberrations are not shared between different stages of the same development path (e.g., gain of chromosome 1 is present in mBc3, but not in the presumed progenitor cells of mBc1) (Figs. 2E,F and 3B,C). This might be caused by a clonal evolution of the malignant cells with the emergence of subclones with distinct chromosomal aberrations [39]. Collectively, we provide evidence for developmental intratumoral heterogeneity of PCNSL.

### Hyperexpanded B cell clones are shared between biopsy- and CSF- but not blood-derived cells

To further study clonal relationships between tissues, we extracted single-cell B cell receptor (scBCR) information from the V(D)J-supplemented scRNA-seq (“Methods”) and identified 4259 cells with a heavy and a corresponding light chain that could be matched to scRNA-seq. Most cells in the malignant clusters mBc1-4 were hyperexpanded clones, while the non-malignant cluster nmBc1-2 predominantly harbored unexpanded cells (single clones) (Fig. 3D–F). Of note, the hyperexpanded clones are spread across all malignant Bc clusters (Fig. 3D–F). The biopsy material showed hyperexpansion of a single malignant clone in each patient (p1_biopsy: ~98% of all cells; p2_biopsy: ~82% of all cells) (Fig. 3G, Additional file 9: Table S7). The CDR3 sequences of the hyperexpanded clones were not related between both patients (Additional file 9: Table S7). All other expanded clones in biopsy were closely related to the hyperexpanded clone with single-nucleotide substitutions within each patient (Additional file 9: Table S7). Notably, we could identify the same hyperexpanded clone in the CSF of patient 1 approximately 1 year after the biopsy during relapse (Fig. 3G, Additional file 9: Table S7). In contrast, we could not identify the hyperexpanded clone in the blood in both patients and there was no relevant clonal expansion in the blood (Fig. 3E,G, Additional file 9: Table S7). We detected a single non-expanded clonotype, located in mBc3, that was shared between the blood and the biopsy material in patient 2 (Additional file 9: Table S7). This might represent a malignant B cell that emigrated from the CNS into the peripheral blood compartment but did not expand. Altogether, prominent hyperexpansion of malignant B cells was restricted to the brain and the CSF. We thus provide evidence that malignant clones are shared between the brain and the CSF, but not between the brain and peripheral blood in PCNSL.

### Hyperexpanded B cell clones show a loss of maturity

Differential expression analysis of the hyperexpanded clones compared to all other clones revealed an elevated expression of tumor promoting factors/oncogenes (*HSPA5*, *PDIA4*, *MANF*, *PPIB*) and a gene associated with malignant B cell clones (*CD63*) [40]. In contrast, BCR activation and signal transduction genes (*BANK1*, *CD37*) and maturity genes (*CD52*, *IGHD*, *MS4A1*) were reduced in the hyperexpanded clones compared to all other clones. CD37, whose expression is related to improved patient survival in peripheral DLBCL, while its loss is a risk factor for therapy resistance with rituximab [41], was also reduced in the hyperexpanded clones (Fig. 3H, Additional file 10: Table S8). Enrichment analysis showed that BCR, RAC1, CXCR4, and ErbB1 signaling pathways were enriched in the genes downregulated in the hyperexpanded clones, while tumor- and cell proliferation-associated pathways (c-Myc, c-Myb, Aurora A, nuclear estrogen receptor alpha pathways) were enriched in upregulated genes of the malignant clones (Fig. 3I, Additional file 11: Table S9). This suggests loss of mature B cell features and increased cell proliferation of the hyperexpanded clones.

### Signs of altered migration in the relapsed clone

To characterize transcriptional changes between the hyperexpanded clone at initial diagnosis and relapse in patient 1 (after high-dose chemotherapy and autologous stem cell transplantation), we performed differential expression analysis (Fig. 3J, Additional file 12: Table S10). The relapsed clone showed an upregulation of *S100A4*, a driver of tumor cell invasion and metastasis [42] and enhanced expression of *CD81*, a tetraspanin molecule, which is crucial for the formation and activation of the B cell coreceptor (CD19–CD21–CD81) complex and has recently been proposed as a novel therapeutic target in B cell lymphomas [43]. We also observed increased expression of *CCL5*, associated with tumor recurrence [44], and an increase of *CCR7*, which controls migration of lymphoma cells into niches [45] (Fig. 3J). As CCL19, the ligand of CCR7, promotes the development of PCNSL through the retention of CCR7 expressing lymphoma cells in the brain [46], the CCR7-CCL19 axis might also play a role in the evasion of malignant B cells from the brain to the CSF. Moreover, we observed a reduced expression of HLA class II molecules (*CD74*, *HLA-DRA*, *HLA-DRB1*, *HLA-DMA*), which might affect the number and function of CD4^+^ T lymphocytes in the tumor microenvironment [47] (Fig. 3J). In summary, the differentially expressed transcripts of the malignant relapsed clone indicated altered migration promoting malignancy compared to the clone before therapy.

### Transcriptional similarity of PCNSL with peripheral B cell lymphomas

We next systematically compared the PCNSL transcriptome with available single-cell data from peripheral Bc lymphomas [15]. We found higher transcriptional correlation between malignant Bc clusters in our dataset (mBc1-4) and published DLBCLs (GC: DLBCL1, DLBCL2; Non-GC: DLBCL3) and GC DLBCL transformed from follicular lymphomas (tFL1, tFL2) and lower correlation between mBc1-4 and follicular lymphomas (FL) (Fig. 3K). In line with previous microarray data [48], this provides evidence for substantial transcriptional overlap between peripheral and central DLBCL. In addition, we systematically compared the chemokine expression between DLBCL and PCNSL (Additional file 4: Fig. S6). We observed that chemokine expression varies considerably between DLBCL and PCNSL, but also between DLBCL samples (GC-derived and non-GC-derived DLBCLs) and within our clusters. Therefore, we could not identify a clear common chemokine pattern that is shared between all PCNSL or all DLBCL and that likely determines the tropism and site specificity of these cells.

### Broad expression of immune checkpoints in the PCNSL microenvironment

**Fig. 4:**
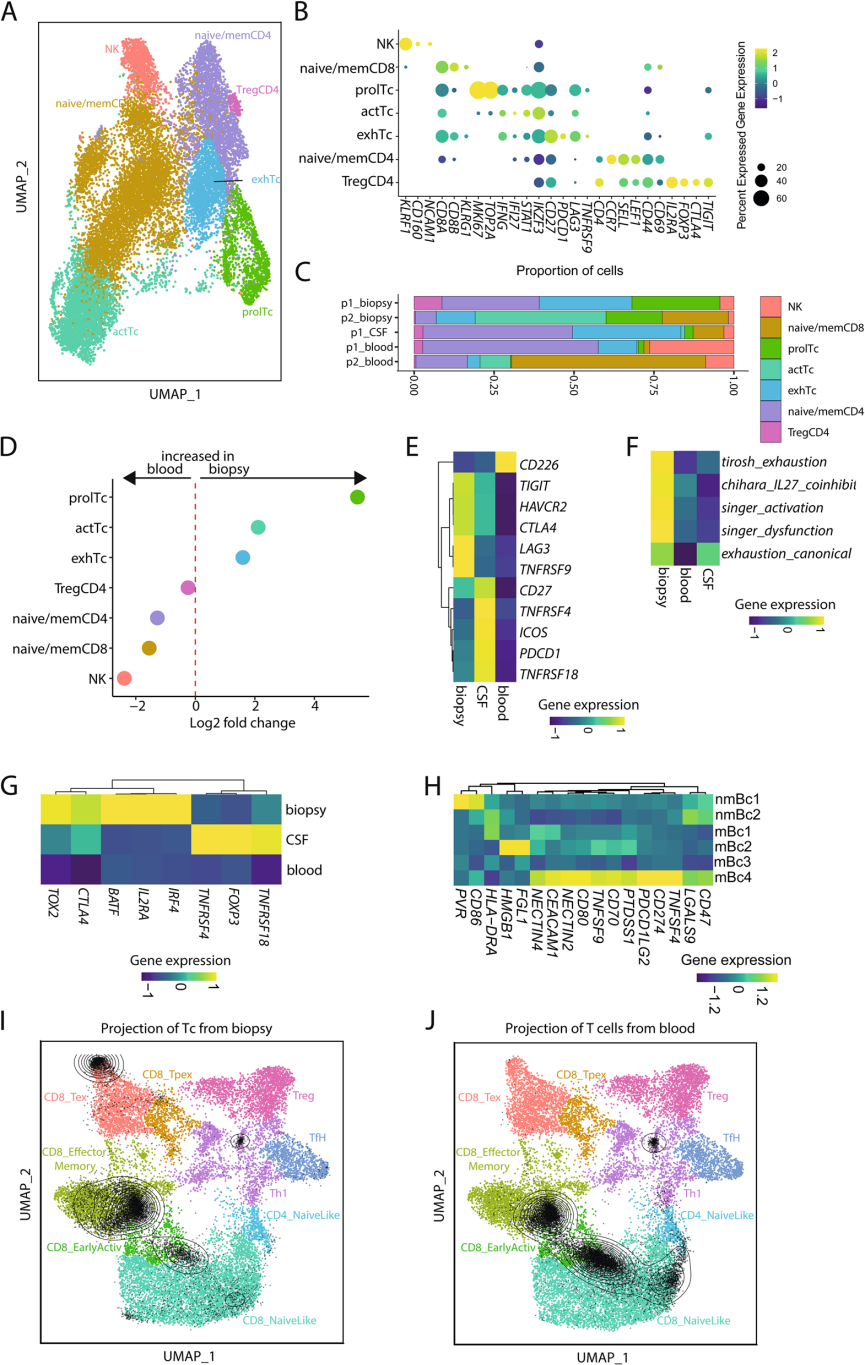
Increased expression of regulatory and T cell exhaustion molecules in the PCNSL microenvironment. **A** UMAP plot of all T cell subclusters, including 17,175 single-cell transcriptomes from 5 samples (patient 1: biopsy, blood, CSF; patient 2: biopsy, blood). **B** Dot plot of T cell markers in T cell subclusters. Color encodes average gene expression and dot size shows the percentage of cells expressing the gene with the threshold of percentage of cells expressing the gene set to 10%. **C** Proportion of T cells split by sample and colorized by cluster name. **D** Comparison of T cell subcluster abundances between biopsy and blood by plotting the log_2_ fold change. **E,F** Gene expression heatmaps of canonical immune checkpoints (**D**), exhaustion profiles from Singer et al. [50], Tirosh et al. [51], Chihara et al. [52] (**F**), and regulatory T cell markers (**G**) in T subclusters. Values were scaled row-wise and color encodes gene expression. Exhaustion signatures are listed in Additional file 14: Table S12. **H** Gene expression heatmap of immune checkpoint ligands in B cell clusters, scaled gene-wise, color encodes gene expression. **I,J** Biopsy-derived T cells (**I**) and blood-derived T cells (**J**) were projected on the latent space of a reference T cell dataset [24]. Gene name - alias: *HAVCR2* - *TIM3*; *PDCD1* - *PD1*; *IRF4* - *MUM1*; *PDCD1LG2* - *PD-L2*; *LGALS9* - *Galectin9*. Abbreviations: NK - natural killer cells; memCD4/CD8 - memory-like CD4^+^/CD8^+^ T cells; naiveCD4/CD8 - naive-like CD4^+^/CD8^+^ T cells; actTc - activated T cells; exhTc - exhausted T cells; TregCD4 - regulatory CD4+ T cells; p1 - patient1; p2 - patient2; CSF - cerebrospinal fluid; mBc - malignant B cells; nmBc - non-malignant B cells; Tex -terminally-exhausted; Tpex - precursor-exhausted; Tfh - CD4+ follicular-helper cells

Based on our FACS data with upregulated PD-1 expression on biopsy-derived Tc, we aimed to further evaluate the expression of immune checkpoints in our scRNA-seq data set. We observed that Tc formed gradients with overlapping signatures rather than distinct subclusters, as we had previously reported [49]. We identified seven sub-clusters (Fig. 4A): NK cells (NK: *KLRF1*, *CD160*, *NCAM1*), CD8^+^ Tc with a naive- and memory-like phenotype (naive/memCD8: *CD8A*, *KLRG1*, *CD44*, *CD69*), proliferating Tc (prolTc: *MKI67*, *TOP2A*), Tc with an activated phenotype with an interferon signature (*IFNG*, *IFI27*, *STAT1*), Tc with an exhausted phenotype (*CD27*, *PDCD1*, *LAG3*, *TNFRSF9*), CD4^+^ Tc with a naive- and memory-like phenotype (naive/memCD4: *CD4*, *CCR7*, *SELL*, *CD44*, *CD69*), and regulatory CD4^+^ Tc (TregCD4: *CD4*, *IL2RA*, *FOXP3*, *CTLA4*) that also expressed markers of T cell exhaustion (*TIGIT*) (Fig. 4B, Additional file 13: Table S11). Biopsy-derived cells featured an increase of prolCD8, actTc, and exhTc and a reduction of NK and naive/memCD4/CD8 compared to blood-derived cells (Fig. 4C,D). This indicated an increase of T cells with proliferating, activated, and exhaustive phenotype in biopsy-derived leukocytes. In line with flow cytometry, nearly all canonical exhaustion molecules, including *TIGIT*, *HAVCR2/TIM-3*, *LAG3*, *CTLA4*, and *PDCD1/PD-1*, were expressed at higher levels in biopsy- or CSF- than in blood-derived cells (Fig. 4E, Additional file 4: Fig. S3). Interestingly, the expression of most markers was divergent between CSF and biopsy, suggesting site specificity in the milieu induced by the tumor cells. By evaluating the expression of several published exhaustion signatures [50–52], we confirmed that biopsy-derived cells showed a higher exhaustion score than blood-derived cells (Fig. 4F, Additional file 14: Table S12). In accordance with flow cytometry, biopsy- and CSF-derived cells also exhibited a stronger regulatory Tc phenotype (*FOXP3*, *IL2RA*, *CTLA4*, *IRF4*) than blood-derived cells (Fig. 4G). We identified several corresponding immune checkpoint ligands in our malignant Bc clusters (e.g., *NECTIN2* and *NECTIN4* bind *TIGIT*; *CEACAM1* binds *HAVCR2/TIM-3; FGL1* binds *LAG3; CD80* binds *CTLA4; CD274/PD-L1* and *PDCD1LG2* bind *PDCD1/PD-1*) (Fig. 4H). Of note, most of these ligands were expressed highest in the mBc4 cluster, indicating that mBc4 induces a particularly immunosuppressive TME.

When projecting biopsy-derived T cells and blood-derived T cells on a recent reference atlas of tumor-infiltrating T cells [24], we observed a large overlap of biopsy-derived T cells with exhausted CD8 T cells (CD8_Tex) (Fig. 4I), which was absent in blood-derived T cells (Fig. 4J). Collectively, we confirmed and extended our flow cytometry findings that showed elevated expression of immune checkpoints in the TME of PCNSL. This suggests a potential of checkpoint inhibitors (CPI) in the treatment of PCNSL and suggests TIGIT, TIM-3, PD-1, CTLA-4, and LAG-3 as promising targets.

### Cellular interactions between PCNSL and its microenvironment reveal immune evasion signaling

**Fig. 5:**
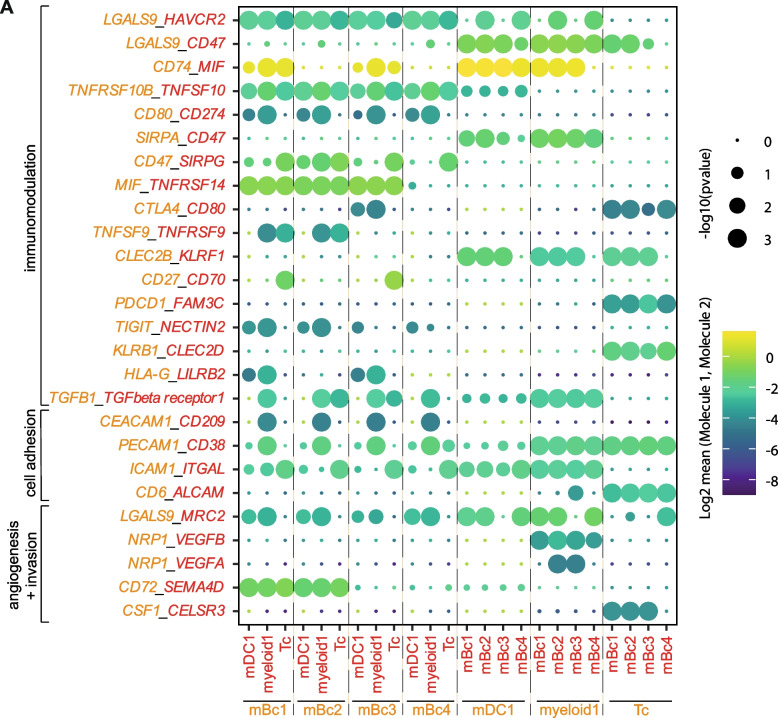
Cellular crosstalk between PCNSL and its microenvironment feature immune evasion signaling. **A** Selected predicted cellular interactions between malignant B cell clusters (mBc1-4) and the immune cell clusters of the tumor microenvironment based on CellPhoneDB [28] (see “Methods”). Circle size represents the *p*-value and color encodes the logarithmic mean of the gene expression of the interacting pairs

To better understand signaling pathways within the tumor micro-milieu, we predicted ligand-receptor pair expression from transcriptome data of biopsy-derived malignant Bc to Tc and myeloid cells (Fig. 5). We identified significant predicted interactions between malignant Bc and immune cells of the TME, e.g., molecules associated with angiogenesis and invasion, including interaction of *NRP1* to *VEGFA* and *VEGFB* between myeloid1 and malignant Bc clusters. Signaling between malignant Bc clusters and their microenvironment also included cell adhesion interactions (e.g., *CD6-ALCAM*, *ICAM1-ITGAL*, *PECAM1-CD38*, and *CEACAM1-CD209*). We identified several immunomodulatory signaling pathways. *CD47* (mBc1-4) and *SIRPA* (myeloid1, mDC1) showed significant interaction, indicating a potential mechanism that protects tumor cells from phagocytosis [53]. Further immunosuppressive signaling between mBc1/3 and myeloid1/mDC1 clusters included interactions between *LILRB2* and *HLA-G*. Blocking of *LILRB2* promotes anti-tumor immunity of myeloid cells [54]. We also identified several known immune checkpoint signaling molecules between Tc and mBc1-4 clusters including *TIGIT*-*NECTIN2*, *CTLA4*-*CD80*, and *HAVCR2*/*TIM-3*-*LGALS9*. Moreover, we observed significant interactions between *KLRB1* (Tc) and *CLEC2D* (mBc1-4), which has recently been described to inhibit killing of glioma cells by T cells [55]. In summary, cellular crosstalk could potentially prevent immune cells from attacking the tumor, thus allowing its immune evasion.

### Transcriptional heterogeneity of malignant B cell clusters is reflected as spatial heterogeneity across patients

We aimed to correlate the observed transcriptional heterogeneity with spatial information. We therefore carried out spatial transcriptomics of the brain biopsy tissues from four patients, including patients 1 and 2 with available matching scRNA-seq data (Additional file 1: Table S1). We obtained an average of 1151 spots per sample with 6925 median genes per spot. In accordance with scRNA-seq and flow cytometry, the biopsy samples showed a broad expression of *CD19*, *MS4A1*/*CD20*, *CD79B*, and *CD27* across the entire tissue (Additional file 4: Fig. S7A-D). T cell transcripts (*CD3E*, *NKG7*) were mostly located in close proximity to the B cells (Additional file 4: Fig. S7A-D), and immunohistochemical stainings of CD20 and CD3 confirmed these findings (Additional file 4: Fig. S7E-G).

**Fig. 6:**
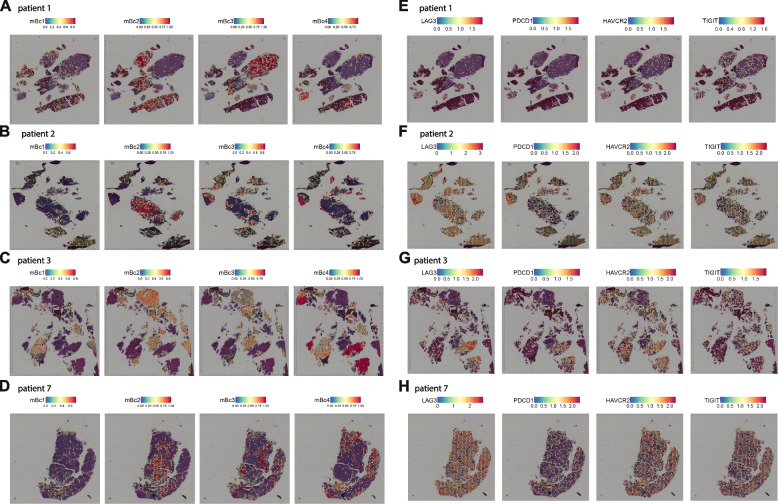
Spatial patterns of malignant B cell clusters reflect their transcriptional heterogeneity across patients. **A–D** Spatial feature plots of the integrated malignant B cell clusters of patients 1 (**A**), 2 (**B**), 3 (**C**), and 7 (**D**). The transcriptional expression is overlaid on top of the tissue histology. The gene expression of the integrated clusters is encoded by color and transparency. **E–H** Spatial feature plots of canonical T cell exhaustion markers of patients 1 (**E**), 2 (**F**), 3 (**G**), and 7 (**H**). The gene expression is encoded by color and transparency. Gene name - alias: *HAVCR2* - *TIM3*; *PDCD1* - *PD1*. Abbreviations: mBc - malignant B cells; nm - non-malignant B cells

In the next step, we computationally integrated our scRNA-seq data with spatial transcriptomics. The malignant B cell clusters, which had been defined by scRNA-seq, displayed areas of focal spatial enrichment in all patients (Fig. 6A–D). Canonical exhaustion markers (*LAG3*, *PDCD1*, *HAVCR2*, *TIGIT*) showed increased expression in tissue areas that were dominated by the mBc4 cluster (Fig. 6E–H). We thus assume that mBc4 induced a stronger immunosuppressive TME than the other malignant B cell clusters. This is in line with our scRNA-seq data of enhanced expression of immune checkpoint ligands, including the ligands of *PDCD1*, *HAVCR2*, and *TIGIT*, in the mBc4 cluster (Fig. 4H). Collectively, we found that transcriptional heterogeneity was replicated as spatial heterogeneity within the tissue and spatially associated one highly malignant B cell cluster with areas of increased immunosuppression.

## Discussion

Conventional histopathology and immunohistochemistry remain the gold standard for the diagnosis and classification of PCNSL. Here, we demonstrate that hematopoietic cells released from CNS biopsy material by the “Whiskey Method” are available for fast-track analysis of suspected PCNSL by flow cytometry and are amenable to high-resolution characterization. DLBCL-type PCNSL was confirmed by histopathology, IgH clonality analysis, and exclusion of extracerebral lymphoma manifestation by bone marrow aspirates in all cases (Additional file 1: Table S1). Our study confirms previous reports investigating brain biopsies after mechanical disaggregation of tissue samples or pure analysis of the rinse fluid with increased proportion of malignant B cells (60–96% of CD45^+^ cells), Ig Kappa, or Lambda light chain restriction (69 - 83%) and high concordance rates of PCNSL diagnosis between flow cytometry and immunohistochemistry (>90%) [56–58]. Due to differences in the study protocols and tissue preparation, direct comparisons with previous approaches are difficult. However, our “Whiskey Method” does not reduce or damage the available tissue material for immunohistopathology and generates sufficient numbers of individual cells for in-depth phenotypical and molecular analysis.

In our cohort, malignant Bc from biopsies displayed an activated memory phenotype characterized by upregulation of CD38 and co-expression of CD27. During B cell differentiation, both CD27 and CD38 are upregulated after activation of Bc in the germinal center (GC) in response to antigen, accompanied by isotype switching recombination and synthesis of immunoglobulins [59, 60]. As the majority of B cells in GCs are usually eliminated during their selection process, the expression of CD27 and CD38, but absence of CD138 suggests that PCNSL cells arise from antigen-experienced late or post GC stages and are prevented from apoptotic cell death, as postulated in earlier studies [23, 36]. Increased CD27 expression could be found in several human B cell malignancies [34] and elevated levels of soluble CD27 were detected in CSF of PCNSLs compared to other brain tumors [35]. Therefore, we propose that CD27 and CD38 should be incorporated into staining protocols for the detection of primary CNS lymphoma. Nevertheless, we note that further markers must be included in clinical practice and validated for a precise classification and differentiation among Bc neoplasms by flow cytometry only [61].

In accordance with a recent scRNA-seq study that analyzed the CSF of PCNSL [62], we detected a transcriptional intratumor heterogeneity of malignant B cells, including differential chemokine expression, and multiple developmental trajectories by using single-cell transcriptomics. As has been described for systemic DLBCL [14], intratumor heterogeneity and subsequent selection of treatment-resistant clones seem to be a driving factor for therapy resistance in PCNSL. We observed altered gene expression in the hyperexpanded clone at relapse, including increase of *CCR7*, which might play a role in the emigration of malignant B cells from the brain to the CSF [46], and *CD81*, which has been identified as a novel immunotherapeutic target for B cell lymphomas [43]. This underscores that single-cell transcriptomics can identify potential new targets for salvage therapies. We also provide evidence that downregulation of HLA class II molecules, which is associated with chromosomal aberrations and copy-number loss at chromosome 6 [12], represents another mechanism of immune evasion in PCNSL.

In line with growing evidence for CPI in PCNSL [13], we discovered broad expression of immune checkpoint molecules in the TME and most of the corresponding ligands in the malignant B cell clusters. The computational prediction of cellular interactions between PCNSL and the TME displayed multiple immunosuppressive interactions. This indicates that PCNSL mediates signals to immune cells in the TME that permit tumor immune evasion. Integration of scRNA-seq data with spatially transcriptomics and immunohistochemistry of tumor tissues revealed distinct and heterogeneous patterns of spatial organization of malignant B cell clusters. We also demonstrate that the topological composition and distribution of different malignant B cell clusters impact the immune micro milieu, facilitating the formation of tumor cell niches with locally enhanced immunosuppression and tumor therapy resistance. These data might help to guide treatment decisions and to develop individualized treatment protocols for patients. Our data support the potential of CPI in the treatment of PCNSL and suggest several immune checkpoint molecules, including CTLA-4, TIGIT, HAVCR2/TIM-3, and LAG-3, as promising targets that should be evaluated in future prospective clinical studies in the treatment of PCNSL for synergistic effects with new B cell targeting approaches including anti-CD79b antibody-drug conjugates, bispecific T-cell engagers, or CAR T-cell therapies [39].

It remains controversial how malignant cells of lymphoid origin reach the nervous tissue. It has been speculated that tumor cells develop in an extracerebral site and migrate to the CNS [40–42]. Although we found a high transcriptional overlap between malignant Bc clusters in peripheral and central DLBCL, our findings do not support a peripheral development of PCNSL since the prominent hyperexpansion of B cells was present in brain- and CSF-derived leukocytes, but not in peripheral blood. We also did not identify a distinct chemokine profile that would favor the migration of peripheral malignant B cells to the CNS. Instead, we speculate that malignant Bc of PCNSL develop within the CNS and that the TME itself fosters the expansion of malignant B cell clones. We detected distinct expressions of chemokines in malignant Bc clusters, which attract a wide range of immune cells, including immunosuppressive leukocytes. This indicated that next to the tumor stroma and resident macrophages/microglia, proliferating neoplastic Bc themselves are heavily shaping their TME.

Our study is limited by the sample size. Therefore, statistical analyses between patients and generalizations entail elements of uncertainty.

## Conclusions

In conclusion, we demonstrate that cells directly released from the biopsy material can support a fast-track detection of PCNSL and a full description of intratumor heterogeneity and the TME at the transcriptional level. Integration of single-cell and spatial transcriptomics can provide further information on the architecture of the intratumor heterogeneity and the PCNSL-TME interface. Expanding this approach to larger patient cohorts will help to design tailored and personalized treatment protocols and to stratify and select more efficacious drug combinations.

## Supplementary Information

**Additional file 1: Table S1.** Patient demographics and sample characteristics.

**Additional file 2: Supplementary Methods**.

**Additional file 3: Table S2.** List of flow cytometry antibodies.

**Additional file 4: Figure S1.** Flow cytometry of B cells. **Figure S2.** Flow cytometry of T cells. **Figure S3.** Flow cytometry of T cell exhaustion markers. **Figure S4.** Histologies of PCNSL and glioblastoma biopsies. **Figure S5.** Patient characteristics of patient 1. **Figure S6.** Chemokines in DLBCL and PCNSL. **Figure S7.** Spatial transcriptomics in PCNSL

**Additional file 5: Table S3.** Technical information about scRNA-seq, scBCR-seq, CITE-seq, and spatial transcriptomics.

**Additional file 6: Table S4.** Top markers of the general clusters.

**Additional file 7: Table S5.** Top markers of the B cell clusters.

**Additional file 8: Table S6.** Chemokine interactions in the mBc clusters.

**Additional file 9: Table S7.** BCR clonotype sequences and frequencies

**Additional file 10: Table S8.** DE genes of hyperexpanded clones vs. all other clones.

**Additional file 11: Table S9.** Enrichment analysis of DE genes from Table S8.

**Additional file 12: Table S10.** DE genes of hyperexpanded clone after vs. before treatment.

**Additional file 13: Table S11.** Top markers of the T cell clusters.

**Additional file 14: Table S12.** Published exhaustion gene signatures.

## Data Availability

All raw single-cell sequencing data, sample, and cluster annotations are available in the Gene Expression Omnibus (GEO) repository: GSE203552 [63]. An interaction version of the single-cell sequencing data created with cerebroApp v1.3 [64] is available at: https://pcnsl.mheming.com.

## Abbreviations

actTc: Activated T cells
BCR: B cell receptor
CSF: Cerebrospinal fluid
DLBCL: Diffuse large B cell lymphoma
exhTc: Exhausted T cells
FL: Follicular lymphomas
GCB: Germinal center B cell
mBc: Malignant B cells
MDSC: Myeloid-derived suppressor cells
mDC1: Myeloid dendritic cells type 1
memCD4/CD8: Memory-like CD4^+^/CD8^+^ T cells
naiveCD4/CD8: Naive-like CD4^+^/CD8^+^ T cells
nmBc: Non-malignant B cells
mBc: Malignant B cells
nmBc: Non-malignant B cells
NK: Natural killer cells
oligo: Oligodendrocytes
p1: Patient 1
p2: Patient 2
PCNSL: Primary central nervous system lymphoma
PLT: Platelets
scBCR: Single-cell B cell receptor sequencing
scRNA-seq: Single-cell RNA-sequencing
Tc: T cells
TME: Tumor microenvironment
Tregs: Regulatory T cells
UMI: Unique molecular identifier
