# Supplementary material for: Intratumor heterogeneity and T cell exhaustion in primary CNS lymphoma
Source: Genome Med. 2022 Sep 24;14:109. doi: 10.1186/s13073-022-01110-1 (PMC9509601; doi:10.1186/s13073-022-01110-1)
Supplement: Supplementary file 2 — Additional file 2: Supplementary Methods. [file 13073_2022_1110_MOESM2_ESM.docx]

**Supplementary Methods**

**Multiparameter flow cytometry - Gating strategy:**

CD45^+^ leukocytes were selected in a CD45/FSC plot. Within this gate, lymphocytes were selected for size in an FSC/SSC plot (forward/sideward scatter channel). For the B cell subtype FACS panel, lymphocytes were subsequently gated on CD19/CD20. B cells (CD19^+^CD20^+^) were further analyzed for the (co-)expression of CD5/CD10, CD38/CD23, CD38/CD27, CD20/CD138, Ig kappa/Ig lambda light chain and PD-L1 (CD274) (Additional file 4: Fig. S1).

For the T cell subtype FACS panels, CD45^+^ leukocytes were gated on CD14/SSC to distinguish between SSC^low^CD14^-^ lymphocytes, SSC^low^CD14^+^ monocytes and SSC^high^CD14^-^ granulocytes. Lymphocytes were further displayed in a CD3/CD56 plot to distinguish between CD3^-^CD56^+^ NK cells, CD3^+^CD56^+^ NKT cells and CD3^+^CD56^-^ T cells. CD3^-^CD56^+^ NK-cells were further split into CD56^bright^CD16^-^ and CD56^dim^CD16^+^ cells and CD3^+^CD56^-^ T cells into CD4^+^ and CD8^+^. HLA-DR expression was then measured on both CD4^+^ and CD8^+^ T cell subsets (Additional file 4: Fig. S2A, C).

For memory phenotype analysis within T cell subsets, lymphocytes were selected on size in an FSC/SSC plot, which were then gated for CD3^+^CD56^-^ T cells and subdivided into CD4^+^ and CD8^+^. The CD4^+^ and CD8^+^ T cells memory subsets were subsequently deﬁned using the following co-expression of markers: naive (Tn; CCR7^+^CD45RA^+^), central memory (Tcm; CCR7^+^CD45RA^-^), effector memory (Tem; CCR7^-^CD45RA^-^), terminally differentiated effector memory (TEMRA; CCR7^-^CD45RA^+^) T cells. Regulatory T cells were determined within CD4^+^ T cells (Treg; CD25^+^CD127^low^) and the expression of the immune checkpoint marker PD1 was analyzed on both CD4^+^ and CD8^+^ T cells. (Additional file 4: Fig. S2B, D)

For the T cell exhaustion panel, lymphocytes were first selected based on size in an FSC/SSC plot, then gated for T cells (CD3^+^CD56^-^) and subsequently for CD4^+^ and CD8^+^ T cell subsets. Within both CD4^+^ and CD8^+^ populations, T cells were analyzed for the co-expression of costimulatory molecule CD28 with the exhaustion markers TIGIT, PD-1, CTLA-4, and Tim-3 (Additional file 4: Fig. S3)

**Further details about generation of single-cell libraries, sequencing, and preprocessing of sequencing data.**

When processing biopsy-derived and blood cells, the total-seq A CITE-seq antibodies listed in Additional file 5: Table S3 were used and purchased from Biolegend. Single-cell suspensions were incubated with CITE-seq antibodies and washed according to the CITE-seq protocol version 2018-09-28 from the New York Genome Center Technology Innovation Lab[[1]](https://sciwheel.com/work/citation?ids=4006944&pre=&suf=&sa=0). Library preparation was performed according to the manufacturer's instructions, using AMPure beads (Beckman Coulter) and half of the resulting cDNA was saved for preparing BCR libraries.

Processing of sequencing data was performed with cellranger v6.1 (10X Genomics) according to the manufacturer’s instructions. Briefly, raw bcl files were de-multiplexed with cellranger mkfastq. Subsequent read alignments and transcript counting was done individually for each sample with cellranger count and default parameters.

**Further details about reconstructing BCR information from 3’ scRNA-seq libraries**

For circularization, cDNA generated from Chromium Single Cell 3’ Library & Gel Bead Kit were end-phosphated with the T4 Polynucleotide Kinase (New England Biolabs), and purified by 0.6x Ampure XP beads (Beckman Coulter). Then 1,000 units of T4 DNA Ligase (New England Biolabs) were added to self-circularize the phosphated cDNA at 16°C for 16 hours. Subsequently, 0.7x Ampure XP beads were used to purify. Remaining linear DNA was digested by 0.9 units/µl RecJf and 0.1 units/µl Lambda Exonuclease (both from New England Biolabs). Circularized cDNA libraries were purified by 0.7x Ampure XP beads. A 5’ race PCR enrichment was performed to enrich the BCR variable region. A size selection was done by 0.5x - 0.8 x Ampure XP beads. Then, the PCR products were phosphated, circulated and linear digested again as above. A PCR with primers read1 and TSO was used to re-linearize the circulated library. PCR products were purified by 0.5x - 0.8 x Ampure XP beads (Beckman Coulter) and libraries were prepared from them, using the Chromium Single Cell 3’ Library Kit v3.1 (10x Genomics). Sequencing was carried out on an Illumina Novaseq6000 with PE150 setup and a NextSeq2000 with 28/8/0/91 sequencing mode. In order to analyze them with cellranger pipelines from 10x Genomics, we converted read 2 into reverse-complement. Processing of modified sequencing data was performed with the cellranger VDJ pipeline v6 (10X Genomics) according to the manufacturer’s instructions.

**References**

[1. Stoeckius M, Hafemeister C, Stephenson W, Houck-Loomis B, Chattopadhyay PK, Swerdlow H, et al. Simultaneous epitope and transcriptome measurement in single cells. Nat Methods. 2017;14:865–8.](https://sciwheel.com/work/bibliography/4006944)
