# Supplementary material for: Intratumor heterogeneity and T cell exhaustion in primary CNS lymphoma
Source: Genome Med. 2022 Sep 24;14:109. doi: 10.1186/s13073-022-01110-1 (PMC9509601; doi:10.1186/s13073-022-01110-1)
Supplement: Supplementary file 4 — Additional file 4: Figure S1. Flow cytometry of B cells. Figure S2. Flow cytometry of T cells. Figure S3. Flow cytometry of T cell exhaustion markers. Figure S4. Histologies of PCNSL and glioblastoma biopsies. Figure S5. Patient characteristics of patient 1. Figure S6. Chemokines in DLBCL and PCNSL. Figure S7. Spatial transcriptomics in PCNSL [file 13073_2022_1110_MOESM4_ESM.pdf]

Additional file 4: Supplementary Figures 1-7

Supplementary Figure 1: Flow cytometry of B cells

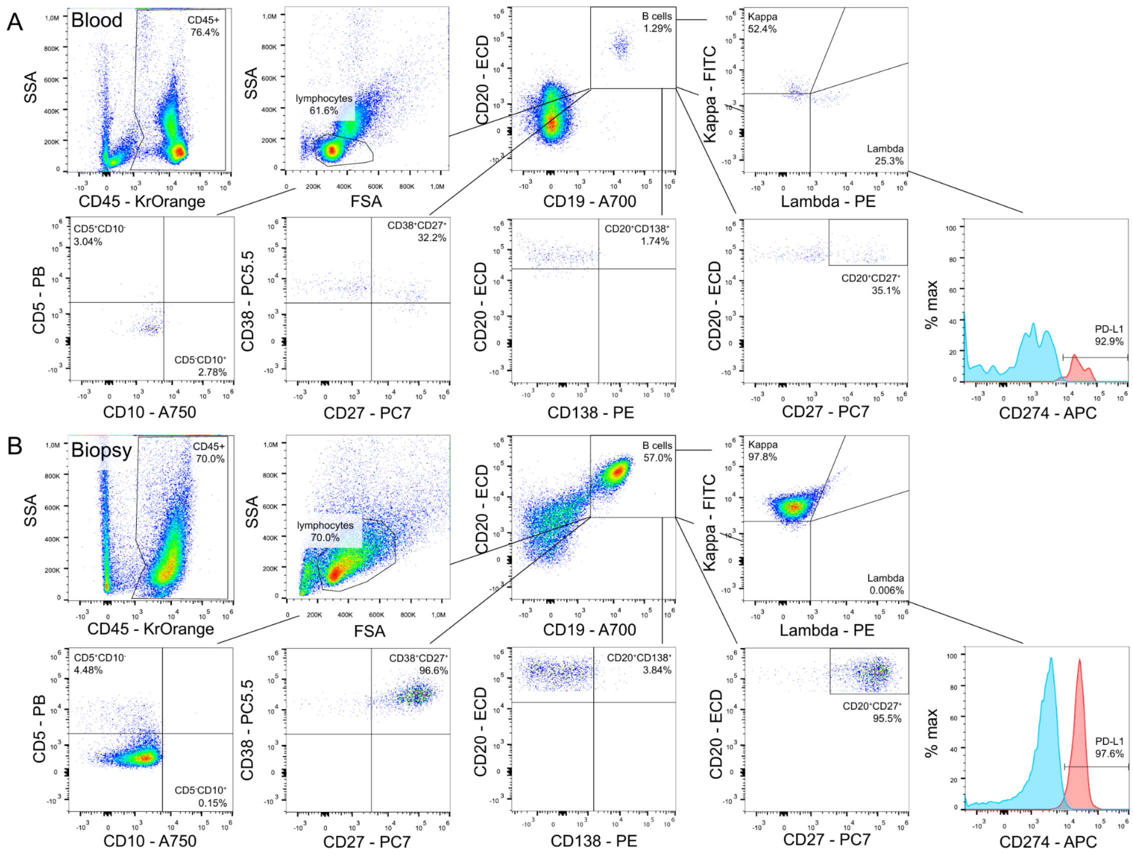

(A-B) Representative flow cytometry staining (p2) of the B cell panel in blood (A) and biopsy (B) material.

Supplementary Figure 2: Flow cytometry of T cells

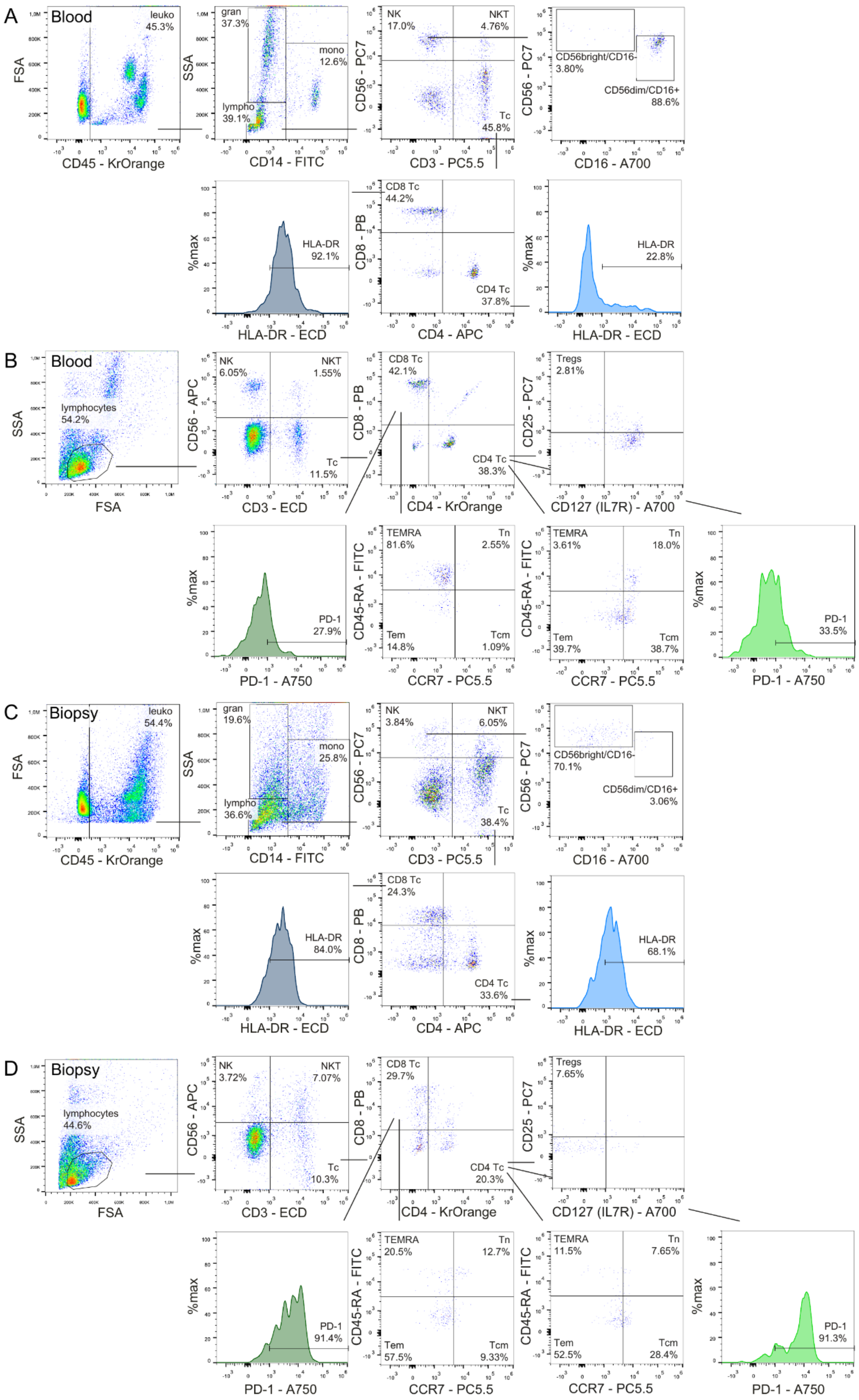

(A-D) Representative flow cytometry staining (p2) of the T cell panel in blood (A-B) and biopsy (C-D) material.

Abbreviations: leuko - leukocytes; gran - granulocytes, lympho - lymphocytes; mono - monocytes; Tc - T cells; Tregs - regulatory T cells, Tcm - central memory T cells; Tem - effector memory T cells, TEMRA - effector memory recently activated T cells

Supplementary Figure 3 : Flow cytometry of T cell exhaustion markers

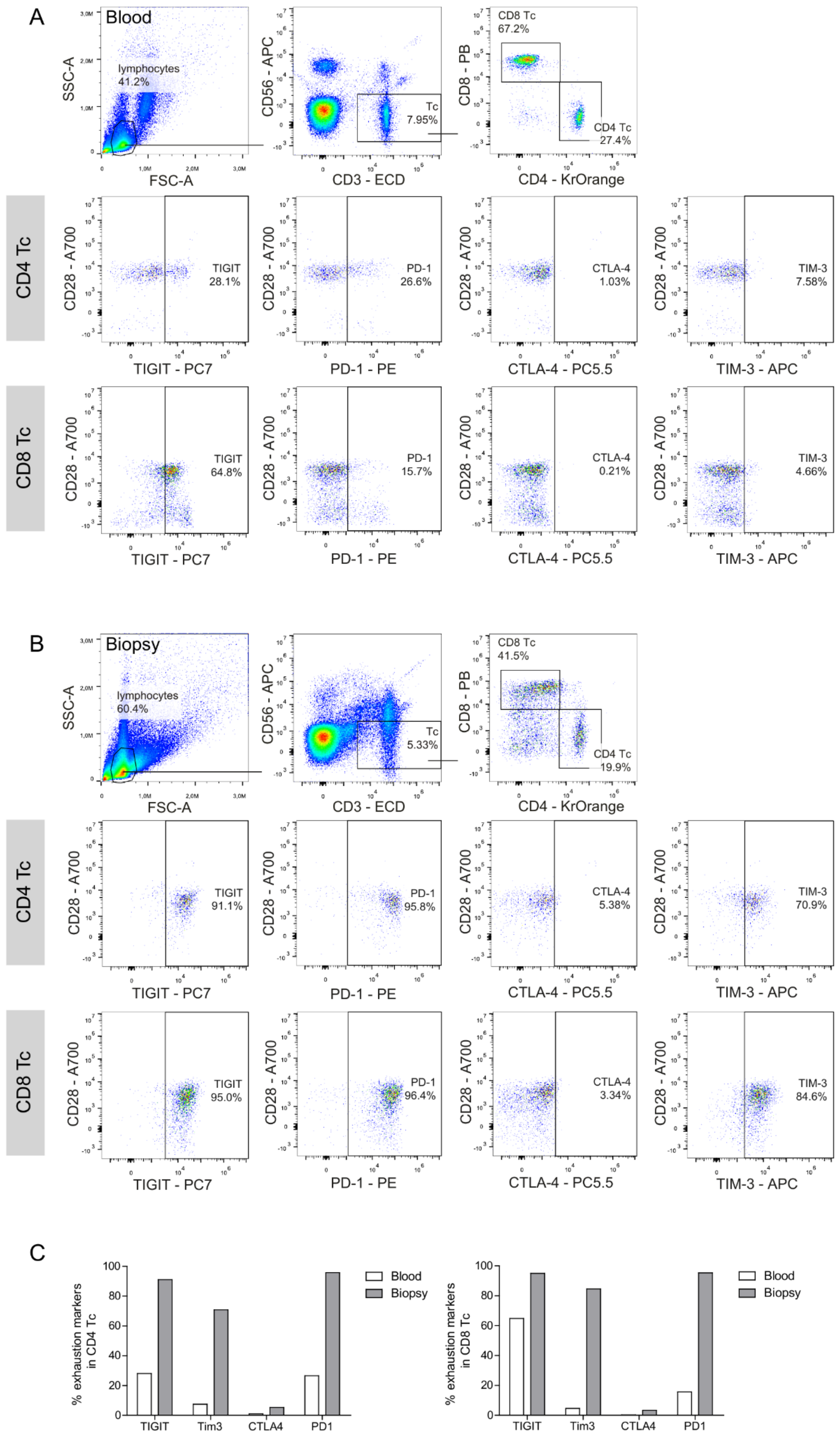

(A-D) Representative flow cytometry staining (p2) of the T cell exhaustion panel in blood (A) and biopsy (B) material.
(C) Bar plots of the percentage of $CD4^+$ or $CD8^+$ T cells expressing T cell exhaustion markers.

Supplementary Figure 4: Histologies of PCNSL and glioblastoma biopsies

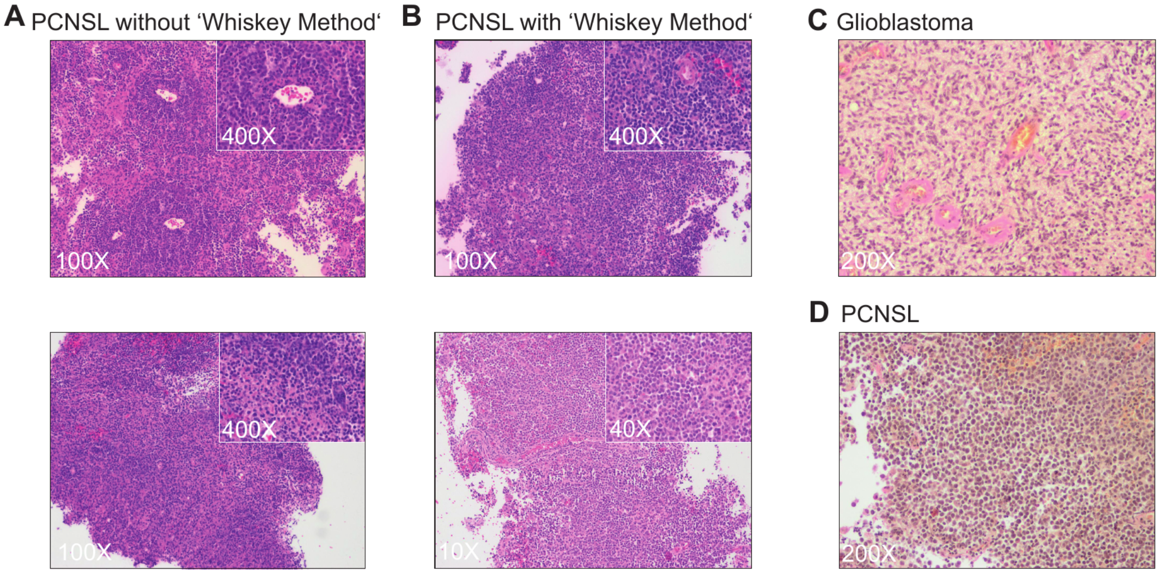

(A-B) Hematoxylin-eosin staining of four PCNSL biopsies . In two cases, the "Whiskey method" was not performed ahead of immunohistochemistry (A) and in the other two cases, the "Whiskey Method" was carried out beforehand (B).

(C-D) Elastica-van-Giesson staining of a glioblastoma (C) and a PCNSL (D)

Supplementary Figure 5: Patient characteristics of patient 1

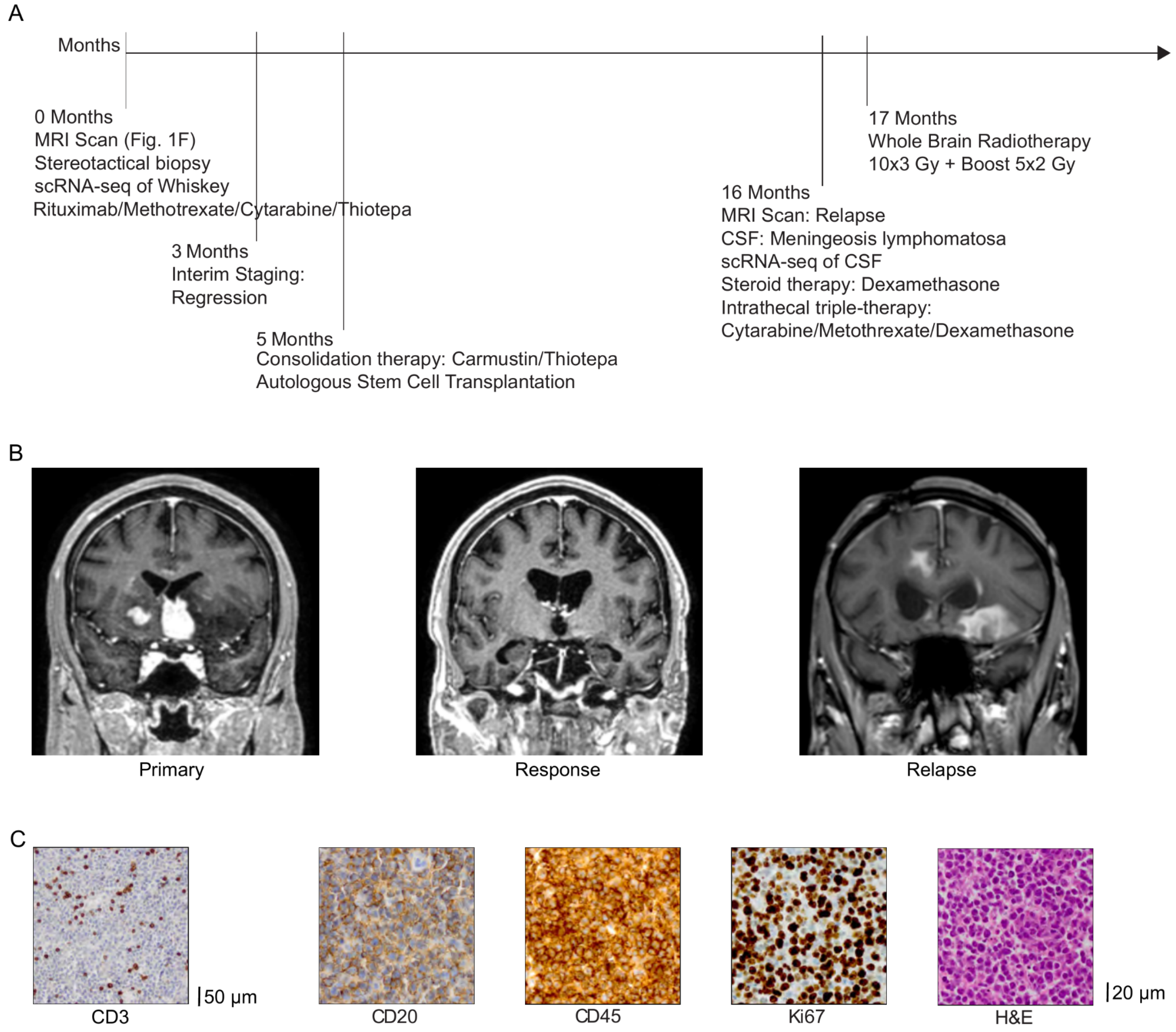

(A) Timeline of clinical course of p1.

(B) Gadolinium enhanced coronal T1 MR images of p1 at primary diagnosis, after response and at relapse.

(C) Immunohistochemistry and hematoxylin-eosin staining of p1.

Supplementary Figure 6: Chemokines in DLBCL and PCNSL

A

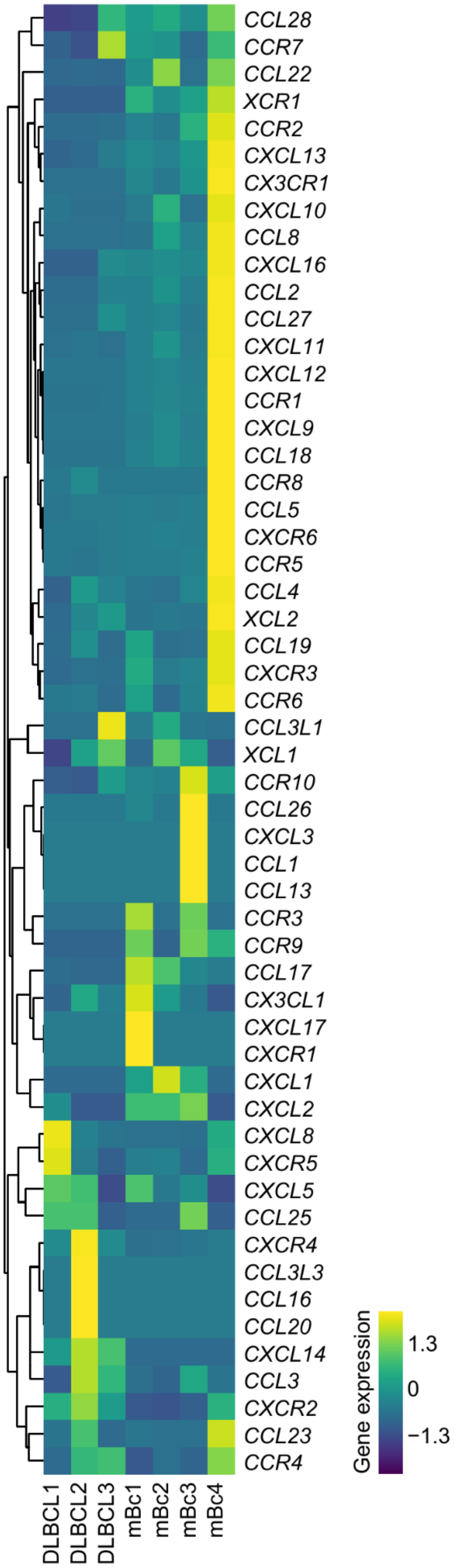

(A) Heatmap of chemokines and their receptors in three DLBCL samples from Roider et al. and malignant B cell clusters from this study. Values were scaled row-wise and color encodes gene expression.

Supplementary Figure 7: Spatial transcriptomics in PCNSL

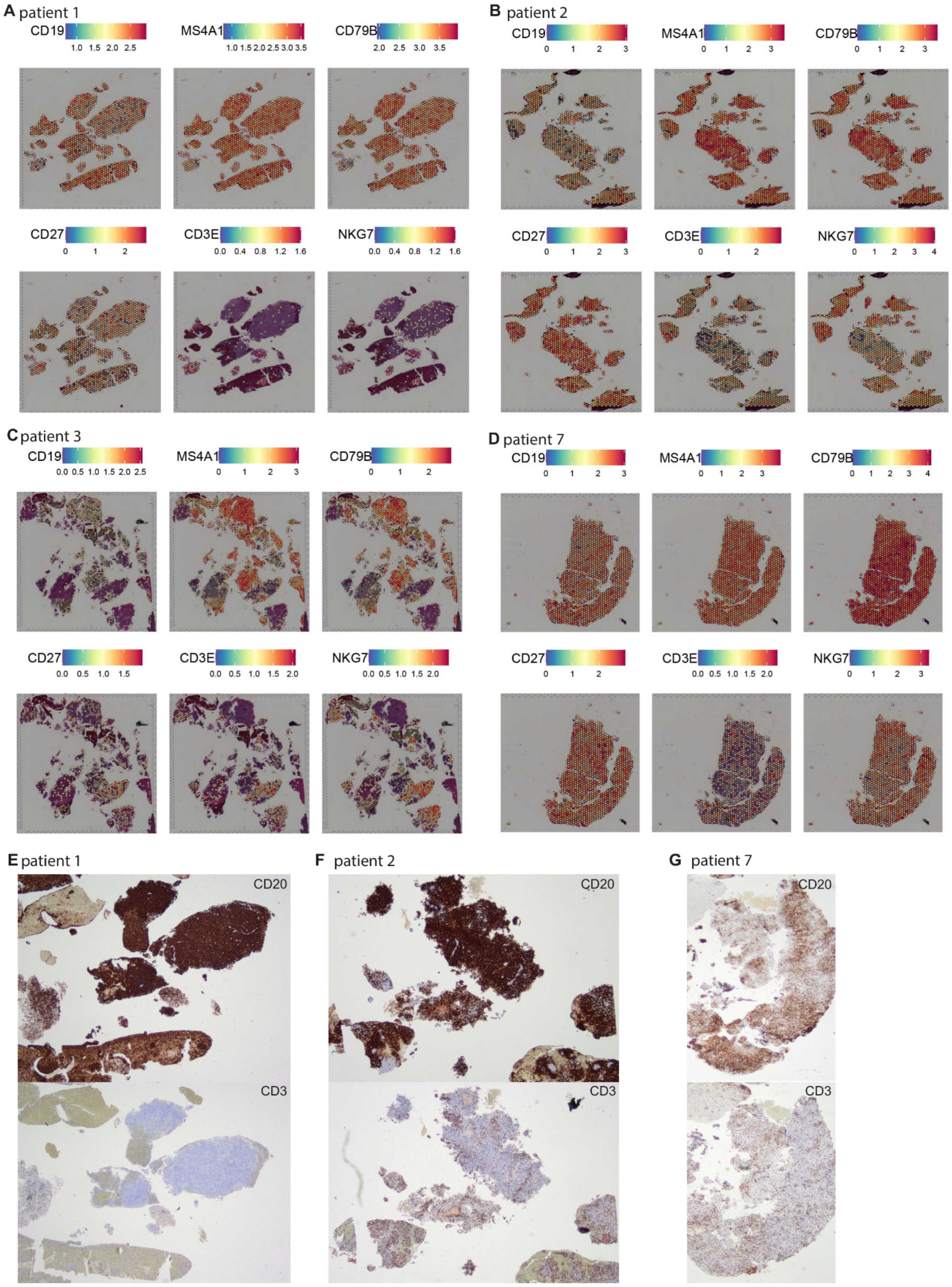

(A-D) Spatial features plots of B cell and T cell markers in p1 (A), p2 (B), p3 (C) and p7 (D). Color and transparency encodes the gene expression.

(E-G) Immunohistochemical staining of B and T cell markers in p1 (E), p2 (F) and p7 (D).
(A-D) Spatial feature plots of the integrated malignant B cell clusters of patients 1 (A), 2 (B), 3 (C) and 7 (D). The transcriptional expression is overlaid on top of the tissue histology. The gene expression of the integrated clusters is encoded by color and transparency.
(E-H) Spatial feature plots of canonical T cell exhaustion markers of patients 1 (E), 2 (F), 3 (G) and 7 (H). The gene expression is encoded by color and transparency.
Gene name - alias: HAVCR2 - TIM3; PDCD1 - PD1
Abbreviations: mBc - malignant B cells; nm - non-malignant B cells
